# Anti-Inflammatory, Antioxidant, and Antifibrotic Effects of Gingival-Derived MSCs on Bleomycin-Induced Pulmonary Fibrosis in Mice

**DOI:** 10.3390/ijms23010099

**Published:** 2021-12-22

**Authors:** Xishuai Wang, Shiyu Zhao, Junhui Lai, Weijun Guan, Yang Gao

**Affiliations:** 1 Department of Animal Genetic Resources, Institute of Animal Science, Chinese Academy of Agricultural Sciences, Beijing 100193, China; 201831070011@mail.bnu.edu.cn (X.W.); zhaoshiyu1234@126.com (S.Z.); dh17744407656@163.com (J.L.); 2 College of P.E and Sport, Beijing Normal University, Beijing 100193, China; 3 Institute of Physical Educational Training, Capital University of Physical Education and Sports, Beijing 100193, China

**Keywords:** lung fibrosis, mesenchymal stem cells, neutrophils, inflammation, oxidative stress

## Abstract

Background: Mesenchymal stem cell (MSC) intervention has been associated with lung protection. We attempted to determine whether mouse gingival-derived mesenchymal stem cells (GMSCs) could protect against bleomycin-induced pulmonary fibrosis. Methods: Mice were divided into three groups: control (Con), bleomycin (Bl), and bleomycin + MSCs (Bl + MSCs). Mice were treated with 5 mg/kg bleomycin via transtracheal instillation to induce pulmonary fibrosis. We assessed the following parameters: histopathological severity of injury in the lung, liver, kidney, and aortic tissues; the degree of pulmonary fibrosis; pulmonary inflammation; pulmonary oedema; profibrotic factor levels in bronchoalveolar lavage fluid (BALF) and lung tissue; oxidative stress-related indicators and apoptotic index in lung tissue; and gene expression levels of IL-1β, IL-8, TNF-α, lysophosphatidic acid (LPA), lysophosphatidic acid receptor 1 (LPA1), TGF-β, matrix metalloproteinase 9 (MMP-9), neutrophil elastase (NE), MPO, and IL-10 in lung tissue. Results: GMSC intervention attenuated bleomycin-induced pulmonary fibrosis, pulmonary inflammation, pulmonary oedema, and apoptosis. Bleomycin instillation notably increased expression levels of the IL-1β, IL-8, TNF-α, LPA, LPA1, TGF-β, MMP-9, NE, and MPO genes and attenuated expression levels of the IL-10 gene in lung tissue, and these effects were reversed by GMSC intervention. Bleomycin instillation notably upregulated MDA and MPO levels and downregulated GSH and SOD levels in lung tissue, and these effects were reversed by GMSC intervention. GMSC intervention prevented upregulation of neutrophil content in the lung, liver, and kidney tissues and the apoptotic index in lung tissue. Conclusions: GMSC intervention exhibits anti-inflammatory and antioxidant capacities. Deleterious accumulation of neutrophils, which is reduced by GMSC intervention, is a key component of bleomycin-induced pulmonary fibrosis. GMSC intervention impairs bleomycin-induced NE, MMP-9, LPA, APL1, and TGF-β release.

## 1. Introduction

Pulmonary fibrosis is characterised by shortness of breath, hypoxemia, and the accumulation of fixed fibrosis that leads to expiratory dyspnoea and is associated with unacceptably high incidence, representing a major healthcare problem with few available treatments [1,2]. Few advances in treatment development have been achieved, and pulmonary fibrosis remains a devastating lung disease. Furthermore, the impact of pulmonary fibrosis is increasing on an annual basis [3,4,5].

Mesenchymal stem cell (MSC) intervention is a promising alternative treatment option for many lung diseases due to its low immunogenicity and potent anti-inflammatory, antifibrotic, and paracrine properties [6,7]. Previous work demonstrated that MSCs exert antifibrotic effects and attenuate experimental pulmonary fibrosis [8,9,10,11]. However, the underlying molecular mechanisms of MSCs effects in pulmonary fibrosis have not been fully elucidated. Gingival-derived mesenchymal stem cells (GMSCs) were isolated and characterised for the first time in 2009, and GMSCs are rich MSC sources and can be harvested at a low cost [12]. The gingiva is a unique masticatory keratinised mucosal tissue characterised by rapid wound healing [13,14]. GMSCs have a neural crest origin and can differentiate into neural cell lineages [15,16]. Hence, GMSCs are used to treat skin diseases, peripheral nerve injuries, and oral and maxillofacial disorders. It is both convenient and economical to separate gingival tissues from patients. We can separate and culture GMSCs from patient gingival tissues and then inject GMSCs into the lung tissues of patients with pulmonary fibrosis. GMSCs may be a novel alternative to autologous stem cell transplantation. Recent work has demonstrated that GMSCs alleviate lung injury [16]. Herein, we attempted to determine whether GMSC intervention could protect against bleomycin-induced pulmonary fibrosis.

Profibrotic mediators are important components in the development of pulmonary fibrosis. Transforming growth factor-β (TGF-β) and platelet-derived growth factor (PDGF) are important profibrotic factors [17,18]. Recent studies have demonstrated that lysophosphatidic acid (LPA) and its receptor lysophosphatidic acid receptor 1 (LPA1) are important causes of pulmonary fibrosis because LPA and LPA1 enhance the proliferation and viability of pulmonary fibroblasts [19]. Antagonists of LPA or gene deletion of its receptor, LPA1, reduced pulmonary fibrosis [20]. Neutrophils are important for the development of pulmonary fibrosis because they produce various proteases, such as neutrophil elastase (NE) and matrix metalloproteinase-9 (MMP-9), which are important profibrotic factors [21,22]. Previous studies demonstrated that MSC intervention reduces the abnormal accumulation of neutrophils in lung tissue [23,24]. MSC intervention may reduce bleomycin-induced neutrophil inflammation in lung tissue. LPA, LPA1, TGF-β, PDGF, NE, and MMP-9 are important profibrotic mediators. However, the physiological roles of these factors in response to GMSC intervention are unknown.

Generally, an increase in the number of inflammatory cells and levels of inflammatory factors caused by bleomycin attracts blood vessels and reaches multiple organs via the circulation, causing vascular, liver, and kidney injuries. However, the influence of bleomycin-induced inflammation of the liver, kidney, and aortic injuries has often been ignored in previous studies. The present study attempted to demonstrate that the inflammatory response caused by bleomycin was systemic and had an impact on multiple organ injuries.

The present preliminary study addressed several important issues, such as simple and effective methods for isolation, culture, and characterisation of mouse GMSCs. Importantly, we attempted to determine whether GMSCs play a prominent role in relieving bleomycin-induced pulmonary fibrosis in mice.

## 2. Methods

### 2.1. Isolation and Culture of GMSCs

GMSCs were separated and cultured as previously described [24]. Mouse gingival tissues were washed 5–7 times with sterile PBS, and as many blood cells as possible were removed. Then, the connective tissues were carefully removed. The remaining gingival tissues were mechanically minced into tiny fragments (1 mm^3^), and the extracellular matrix of the minced tissues was dissociated using 2 mg/mL collagenase type I and 4 mg/mL trypsin II at 37 °C for 70 min. Complete DMEM/F12 supplemented with 10% foetal bovine serum (FBS) was used to immediately neutralise the enzymatic reaction. The resulting cell suspension was filtered through a 100 µm mesh sieve and then centrifuged at 1200 r/min for 10 min. Then, the cell pellet was resuspended in a complete DMEM/F12 medium containing 10% FBS and 2 mM L-glutamine, seeded into 6-well plates (10^4^ cells per well) and cultured at 37 °C in a humidified incubator with 5% CO_2_. GMSC were cultured in a complete medium. All information about the induction medium is listed in Supplementary Material 1.

To minimise heterogeneity, gingival tissues were isolated from six individual mice. Cryopreservation of MSCs was essential for further experiments. When GMSCs reached 90% confluence, the cells were digested and harvested in 15-mL sterile centrifuge tubes. The cell pellet was resuspended in a freezing medium composed of 50% FBS, 40% DMEM/F12, and 10% DMSO. One millilitre of the resulting suspension (1 × 10^6^ cells/mL) was transferred into 1.8-mL sterile cryogenic tubes followed by storage at −80 °C overnight, and GMSCs were transferred to liquid nitrogen tanks for long-term storage.

### 2.2. Immunofluorescence Staining

We performed immunofluorescence staining as previously described [25]. After GMSCs in the third passage reached 40–50% confluence, the cells were fixed in ice-cold PBS containing 4% paraformaldehyde (PFA) for 15 min at room temperature and washed 3 × 5 min. GMSCs were then permeabilised with 0.1% Triton X-100 for 30 min at room temperature and washed 3 × 5 min. Nonspecific binding sites were blocked by incubating cells with 10% sheep serum for 30 min at room temperature. After blocking, GMSCs were incubated overnight at 4 °C with the following primary antibodies: FITC-rabbit Sox-2, OCT-4, CD73, CD90, CD105, CD34, and CD45 antibodies (1:200, Abcam, Cambridge, MA, USA). After incubation, GMSCs were washed 3 times and incubated in PBS containing a FITC-conjugated goat anti-rabbit secondary antibody (1:100, Santa Cruz, CA, USA) for 2 h at 37 °C in the dark and washed 3 × 5 min. GMSCs were counterstained with 1 µg/mL DAPI in the dark for 15 min at room temperature. Finally, the fluorescence signals were captured under a fluorescence microscope (Nikon TE-2000-E, Tokyo, Japan).

### 2.3. Multi-Differentiation Potential of GMSCs

#### 2.3.1. Osteogenic Differentiation of GMSCs

GMSCs were seeded into 12-well plates (2.0 × 10^5^ cells/well). When third-generation GMSCs reached 30–40% confluence, the medium was replaced with an osteogenic differentiation medium. After 21 days of culture and differentiation, Alizarin Red staining was performed to detect calcium deposition. All information about the induction medium is listed in Supplementary Material 1.

#### 2.3.2. Adipogenic Differentiation of GMSCs

As described above, third-generation GMSCs reached 30–40% confluence were cultured in adipogenic differentiation medium. After 14 days, Oil Red O staining was performed to evaluate lipid accumulation.

#### 2.3.3. Chondrogenic Differentiation of GMSCs

For chondrogenic differentiation, third-generation GMSCs were cultured in a chondrogenic medium for 21 days, and Alcian blue staining was performed to confirm chondrogenic differentiation.

#### 2.3.4. Neuroblastic Differentiation of GMSCs

When 40–50% confluence was achieved, GMSCs were exposed to a neuroblastic differentiation medium, which was replaced with 50% fresh medium every 2 days. On day 21 of differentiation and culture, morphological changes were captured under an inverted microscope.

### 2.4. Clone Formation Assay of GMSCs

Third-generation GMSCs were dissociated, and a series of dilutions were used to produce a single-cell suspension. After counting, 100 cells were inoculated into a cell culture dish. GMSCs were stained with Giemsa, and cellular morphology was observed under an inverted microscope after 7 days.

### 2.5. Experimental Design

All protocols used in this study were approved by the Animal Experimental Welfare of the Institute of Animal Science, Chinese Academy of Agricultural Sciences (Beijing, China). All experiments were performed in accordance with the Animal Experimental Welfare of the Institute of Animal Science, Chinese Academy of Agricultural Sciences and the Guide for the Care and Use of Laboratory Animals published by the US National Institutes of Health. Thirty female ICR mice (8 weeks old, 18–21 g) were provided by Beijing HFK Biotechnology Co., Ltd. (Beijing, China). The authors have read the ARRIVE guidelines, and this study was performed in compliance with the ARRIVE guidelines. All mice were anaesthetised via intraperitoneal injection of pentobarbital (50 mg/kg). Mice were euthanised with isoflurane.

Pulmonary fibrosis was established by transtracheal instillation of 5 mg/kg bleomycin as previously described [26,27]. Thirty mice were randomly assigned into three groups: (1) control (Con) mice received an equivalent volume of normal saline via transtracheal instillation; (2) bleomycin (Bl) mice received 5 mg/kg bleomycin via transtracheal instillation; and (3) bleomycin + MSC (Bl + MSC) mice received 5 mg/kg bleomycin via transtracheal instillation and 1 × 10^6^ GMSC via transtracheal instillation. Undifferentiated GMSCs (passage 5) were thawed in a 37 °C water bath and washed in PBS to remove the cell freezing solution prior to injection. GMSCs were then passed through a 100-μm cell strainer to remove cell clumps. GMSCs were counted and resuspended in PBS immediately prior to injection. Mice were administered 1 × 10^6^ GMSCs via transtracheal instillation. Mice were injected with GMSCs once a week for 4 weeks. Mice were sacrificed after 4 weeks. Each group included 10 mice.

### 2.6. Histopathology

For histological analysis, the lung, kidney, liver, and aortic tissue samples were fixed in 4% PFA, dehydrated, and embedded in paraffin as previously described [28]. Five-micrometre-thick sections were stained with Mason, Sirius Red, and haematoxylin eosin (HE). The images were captured using an inverted microscope. Neutrophil content, lung injury score, kidney injury score, liver injury score, and pulmonary fibrosis score are provided in Supplementary Material 2.

### 2.7. Collection of Bronchoalveolar Lavage Fluid (BALF) and Tissue Samples

The mice were euthanised using isoflurane (blood collection through the abdominal vein). Two millilitres of ice-cold physiological saline was used for total lung lavage via the tracheal cannula. The lungs were flushed four times, and the returned fluid was collected and centrifuged at 3000 r/min for 12 min at 4 °C. The supernatant was stored at −80 °C for subsequent analysis of cytokine levels. After BALF collection, perfusion or lavage of the organ was performed using physiological saline. Finally, the liver, kidney, and aortic tissues were collected.

After sampling, the remaining lung, liver, kidney, and aortic tissues were preserved in liquid nitrogen containers and immediately placed in an incubator filled with liquid nitrogen. Then, the tissues were transferred to liquid nitrogen tanks for long-term preservation. Samples of the lung, liver, and kidney tissues of appropriate size were fixed in 4% paraformaldehyde for 24 h.

### 2.8. Determination of Pulmonary Oedema

To quantify the degree of pulmonary permeability, the wet-dry weight ratio (W/D) of lung tissues and the protein content in BALF were detected. Bibulous paper was used to absorb the liquid and blood from the surface of the lung tissue, and the wet weight of the lung tissue was determined. The lung tissue was dried to reach a stable dry weight. W/D = wet weight of the lung tissue/dry weight of the lung tissue. A Bio-Rad protein assay kit was used to assay the protein concentration in BALF.

### 2.9. Determination of Oxidative Stress Index in Lung Homogenates

Malondialdehyde (MDA), superoxide dismutase (SOD), myeloperoxidase (MPO), and glutathione S-transferase (GSH) levels in lung homogenates were detected by spectrophotometry. We measured the conjugation of 1-chloro-2,4-dinitrobenzene (CDNB) to evaluate GST activity [29]. The Bradford method was used to evaluate MDA activity [30]. We measured MPO and SOD, as previously described [31,32].

### 2.10. Cytokines

The levels of IL-1β, CXCL-1, IL-8, TNF-α, TGF-β, MMP-9, IL-10, and NE activity in BALF were measured using mouse ELISA kits (Neobioscience) according to the manufacturer’s instructions.

### 2.11. qRT–PCR

Total RNA extracted from the lung tissue was used as a template for cDNA synthesis. We performed qRT–PCR as previously described [33]. GAPDH was used as a housekeeping gene. Information on mouse PCR primer sequences is shown in Supplementary Material 3.

### 2.12. Statistical Analysis

GraphPad Prism 9 software was used to perform one-way ANOVA and to plot the graphs. The significance threshold was set to *p* < 0.05. All data are expressed as the mean ± SEM.

## 3. Results and Discussion

### 3.1. Biological Characteristics of Mouse GMSCs

**Figure 1:**
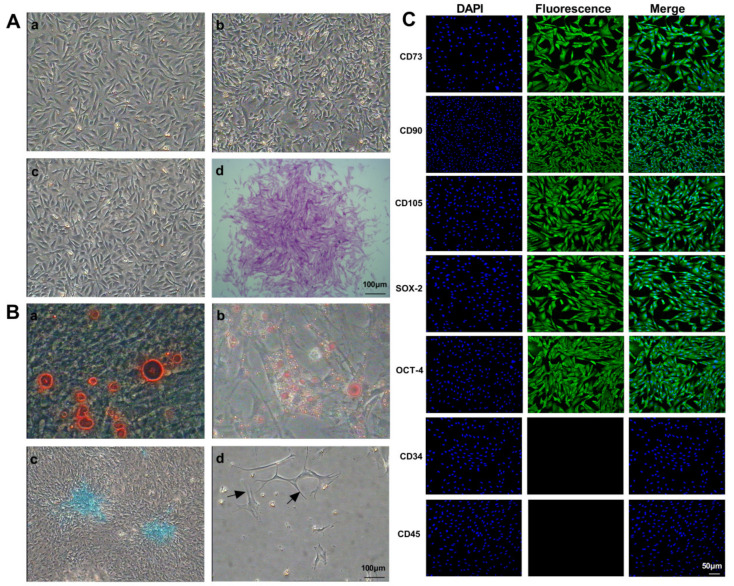
Biological characteristics of mouse GMSCs. (**A**) Morphology of GMSCs. (A-a) Cells that were initially round or irregularly shaped began to attach to the surface after primary culture for 24 h. (A-b) Generally, after 2–3 passages, morphological differences disappeared, and all cells exhibited a fibroblast-like morphology. (A-c) Typically, primary cells proliferated to 80–90% confluence after culture for 5–7 days; then, GMSCs proliferated rapidly and could be subcultured every 1–2 days. (A-d) Spindle-shaped GMSCs gave rise to colonies. (**B**) Detection of the multilineage differentiation potential of mouse GMSCs. (B-a) Detection of Alizarin Red staining. (B-b) Detection of Oil Red O staining. (B-c) Detection of Alcian Blue staining. (B-d) Morphological changes in GMSCs under neurogenic induction conditions after 21 days. Black arrows indicate that GMSCs grew numerous branches and formed many synapses on the cell surface. (**C**) Immunofluorescence staining for GMSC surface antigens, including CD73, CD90, CD105, Sox-2, OCT-4, CD34, and CD45.

The morphology of mouse GMSCs was determined at various passages (P1, P15, and P25) (Figure 1A(a–c)). Spindle-shaped GMSCs gave rise to colonies, suggesting that GMSCs possessed colony formation ability (Figure 1A(d)). We next determined the multilineage differentiation potential of GMSCs. Under adipogenic, osteogenic, chondrogenic, and neuronal induction conditions for 14–21 days, GMSCs were induced to differentiate into adipocytes, osteoblasts, chondrocytes, and neuroblasts, respectively. Clear morphological differences between differentiated and undifferentiated GMSCs were observed. Under osteogenic induction conditions, numerous calcium nodules appeared in the cytoplasm, and the results of Alizarin Red staining indicated positive reactivity of calcifying nodules in differentiated GMSCs (Figure 1B(a)). Under adipogenic induction conditions, cellular morphology changed from spindle-like to oblate in shape, and numerous lipid droplets were positively stained by Oil Red O staining in the cytoplasm (Figure 1B(b)). After incubation in a chondrogenic medium for 21 days, a clear morphological change was detected, and GMSCs were positively stained with Alcian Blue (Figure 1B(c)). Under neurogenic induction conditions for 21 days, the shape of GMSCs changed from spindle-shaped to multipolar and stellate morphology, and GMSC growth resulted in the formation of numerous branches and synapses on the cell surface (Figure 1B(d)). Immunofluorescence staining for GMSC surface antigens, including CD73, CD90, CD105, Sox-2, OCT-4, positive, while CD34 and CD45 were not expressed (Figure 1C). The result of flow cytometry showed that GMSCs surface antigens aSMA and FSP1 were positively expressed (Supplementary Material 4).

### 3.2. Assessment of Lung Injury and Neutrophil Infiltration

**Figure 2:**
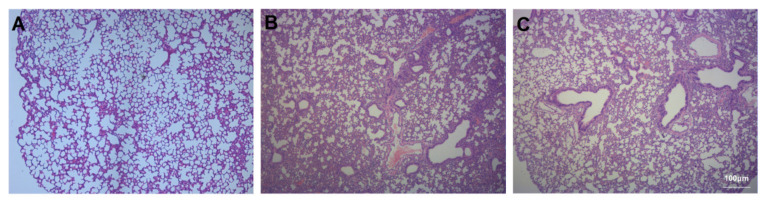
Histological assessment of the lung tissue: (**A**) Con group; (**B**) Bl group; (**C**) Bl + MSC group (**A**–**C**, 100× magnification).

Histological assessment of lung tissue samples from the three groups revealed the degree of lung injury, inflammatory infiltration, and interstitial oedema. Compared to the lung tissue of Con mice (Figure 2A), the degree of lung injury, inflammatory infiltration, and interstitial oedema increased after bleomycin instillation (Figure 2B). Compared to the Bl group, the degree of lung injury, inflammatory infiltration, and interstitial oedema were attenuated after GMSC intervention (Figure 2C).

**Figure 3:**
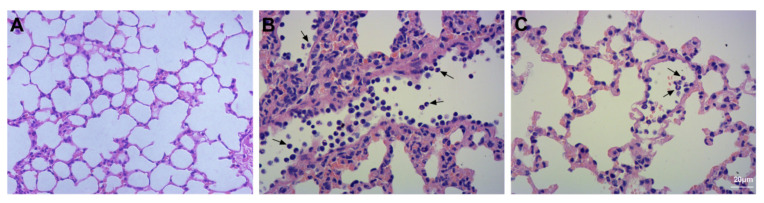
Detection of neutrophil infiltration: (**A**) Con group; (**B**) Bl group; (**C**) Bl + MSC group; Black arrows indicate neutrophils (**A**–**C**, 400× magnification).

The images revealed the contents of neutrophils in the lung tissue. Neutrophils were not detected in lung tissue in the Con group (Figure 3A). Bleomycin instillation led to severe neutrophil infiltration compared to that in the Con group (Figure 3B). Compared to the Bl group, GMSC intervention attenuated neutrophil infiltration in lung tissue (Figure 3C).

### 3.3. Assessment of Pulmonary Fibrosis

**Figure 4:**
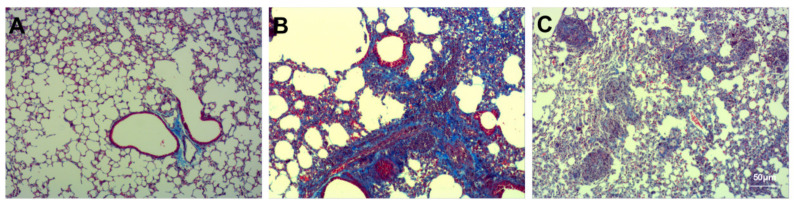
Detection of pulmonary fibrosis: (**A**) Con group; (**B**) Bl group; (**C**) Bl + MSC group; Mason staining revealed the degree of pulmonary fibrosis. (**A**–**C**, 200× magnification).

**Figure 5:**
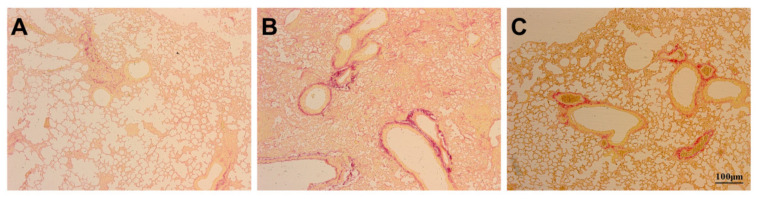
Detection of pulmonary fibrosis in the airway wall: (**A**) Con group; (**B**) Bl group; (**C**) Bl + MSC group; Sirius Red staining revealed collagen fibre deposition in the lung. (**A**–**C**, 100× magnification).

Masson staining revealed the degree of pulmonary fibrosis. Compared to the Con group (Figure 4A), the degree of pulmonary fibrosis in the airway wall increased after bleomycin instillation (Figure 4B). Compared to the Bl group, GMSC intervention attenuated the degree of pulmonary fibrosis (Figure 4C). Sirius Red staining revealed collagen fibre deposition in the airway wall. Comparison to the Con group (Figure 5A) indicated that bleomycin instillation increased collagen fibre deposition in the airway wall (Figure 5B). Compared to the Bl group, GMSC intervention attenuated collagen fibre deposition in the airway wall (Figure 5C).

### 3.4. Assessment of Apoptosis

**Figure 6:**
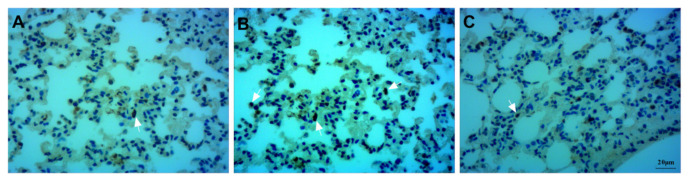
Detection of apoptosis in lung tissue: (**A**) Con group; (**B**) Bl group; (**C**) Bl + MSC group; TUNEL staining revealed apoptosis in lung tissue. White arrows indicate apoptotic cells (**A**–**C**, 400× magnification).

TUNEL staining revealed apoptosis in the lung tissue. A small number of the cells were apoptotic in the Con group (Figure 6A), and numerous apoptotic cells were detected after bleomycin instillation (Figure 6B). GMSC intervention notably attenuated the number of apoptotic cells (Figure 6C).

### 3.5. Assessment of Liver Injury

**Figure 7:**
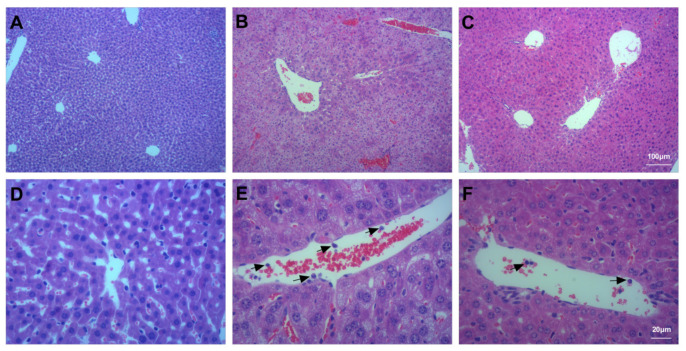
Histological assessment of the liver tissue: (**A**) Con group; (**B**) Bl group; (**C**) Bl + MSC group; (**D**) Con group; (**E**) Bl group; (**F**) Bl + MSC group. Black arrows indicate neutrophils (**A**–**C**, 100× magnification; **D**–**F**, 400× magnification).

The liver lobules of mice in the Con group were intact and clear; the cells were properly arranged, and intercellular regions were free of oedema; the liver sinusoids were clear and regular, and there were no symptoms of injury (Figure 7A,D). The liver lobules of mice in the Bl group were severely damaged; the liver cells were swollen, and the intercellular matrix disappeared; numerous neutrophils were detected (Figure 7B,E). The liver lobules of mice in the Bl + MSC group exhibited significantly less structural damage to the liver tissue, displaying clearer liver lobules and reduced neutrophil infiltration (Figure 7C,F).

As shown in Table 1, bleomycin instillation notably increased markers of liver damage, including ALT (*p* < 0.05) and AST (*p* < 0.05), compared to Con, and GMSCs notably attenuated the levels of ALT (*p* < 0.05) and AST (*p* < 0.05) compared to Bl. As shown in Supplementary Material 2, Bl notably increased the liver injury score compared to Con (*p* < 0.05), and GMSC intervention notably attenuated the liver injury score (*p* < 0.05) compared to Bl.

### 3.6. Assessment of Kidney Injury

**Figure 8:**
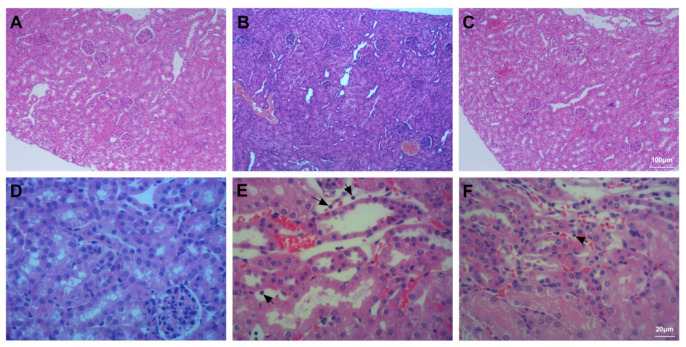
Histological assessment of the kidney tissue: (**A**) Con group; (**B**) Bl group; (**C**) Bl + MSC group; (**D**) Con group; (**E**) Bl group; (**F**) Bl + MSC group. Black arrows indicate neutrophils (**A**–**C**, 100× magnification; **D**–**F**, 400× magnification).

The kidney tissues of mice in the Con group were intact and clear; the cells were properly arranged, and the intercellular substance was free of oedema; there were no symptoms of injury (Figure 8A,D). The kidneys of mice in the Bl group were severely damaged; the cells were swollen, and intercellular substances disappeared; these changes were accompanied by neutrophil and haemocyte infiltration (Figure 8B,E). The kidneys of mice in the Bl + MSC group exhibited significantly less structural damage to the kidney tissue than those in the Bl group, with clearer nephrons reduced inflammatory cell infiltration (Figure 8C,F).

As shown in Table 1, Bl notably increased markers of kidney damage (Cre and BUN) compared to those in the Con group (*p* < 0.05). Furthermore, GMSC intervention notably attenuated the levels of Cre (*p* < 0.05) and BUN (*p* < 0.05) compared to those in the Bl group. As shown in Supplementary Material 2, Bl notably increased the kidney injury score compared to Con (*p* < 0.05), and GMSC intervention notably attenuated the liver injury score (*p* < 0.05) compared to Bl.

### 3.7. Assessment of Aortic Injury

**Figure 9:**
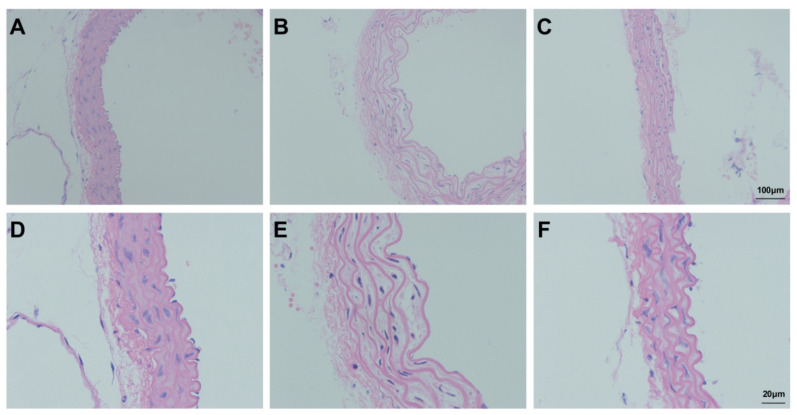
Morphological analysis of the aorta: (**A**) Con group; (**B**) Bl group; (**C**) Bl + MSC group; (**D**) Con group; (**E**) Bl group; (**F**) Bl + MSC group (**A**–**C**, 100× magnification; **D**–**F**, 400× magnification).

In the Con group, the aorta was uniformly stained and regularly arranged; the endothelium was smooth and organised, and the elastic fibres exhibited a regular wavy shape (Figure 9A,D). After bleomycin instillation, the endothelium was not smooth or regular, and elastic fibres of the media became sparse and lost their regular wavy shape (Figure 9B,E). Comparison to the Bl group indicated that the shape of endothelial elastic fibres and their distribution were improved after GMSC administration; however, the fibres did not completely return to their normal form (Figure 9C,F). As shown in Supplementary Material 2, Bl notably increased aortic intima media thickness compared to Con (*p* < 0.05), and GMSC intervention notably attenuated aortic intima media thickness compared to Bl.

### 3.8. Assessment of mRNA Expression in Lung Tissue

**Figure 10:**
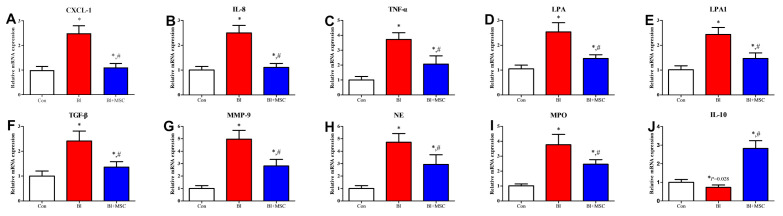
Effects of GMSCs on the gene expression levels of inflammatory and fibrotic factors. Assessment of the mRNA expression levels of the inflammatory factors CXCL-1 (**A**), IL-8 (**B**), TNF-α (**C**), LPA (**D**), LPA1 (**E**), TGF-β (**F**), MMP-9 (**G**)**,** NE (**H**)**,** MPO (**I**) and IL-10 (**J**) in lung tissue. * *p* < 0.05 indicates significant differences versus the Con group; ^#^ *p* < 0.05 indicates significant differences versus the Bl group. Values are expressed as the mean ± SEM. Abbreviations: IL, interleukin; TNF, tumour necrosis factor; CXCL, C-X-C motif chemokine ligand; TGF, transforming growth factor; LPA, lysophosphatidic acid; LPA1, lysophosphatidic acid receptor 1; MMP, matrix metalloproteinase; NE, neutrophil elastase; MPO, myeloperoxidase. White bars indicates Con group, red bars indicates Bl group, blue bars indicate Bl + MSC group.

Bleomycin instillation notably increased the mRNA expression levels of the neutrophil chemoattractant CXCL-1 (*p* < 0.05; Figure 10A), IL-8 (*p* < 0.05; Figure 10B), and TNF-α (*p* < 0.05; Figure 10C), and profibrotic factors LPA (*p* < 0.05; Figure 10D), LPA1 (*p* < 0.05; Figure 10E), TGF-β (*p* < 0.05; Figure 10F), MMP-9 (*p* < 0.05; Figure 10G), NE (*p* < 0.05; Figure 10H), and MPO (*p* < 0.05; Figure 10I) and notably attenuated mRNA expression levels of the anti-inflammatory factor IL-10 (*p* < 0.05; Figure 10J) compared to those in the Con group. GMSC intervention notably increased mRNA expression levels of IL-10 and notably attenuated mRNA expression levels of CXCL-1 (*p* < 0.05), IL-8 (*p* < 0.05), TNF-α (*p* < 0.05), LPA (*p* < 0.05), LPA1 (*p* < 0.05), TGF-β (*p* < 0.05), MMP-9 (*p* < 0.05), NE (*p* < 0.05), and MPO (*p* < 0.05) compared to those in the Bl group.

### 3.9. Assessment of Pulmonary Oedema

**Figure 11:**
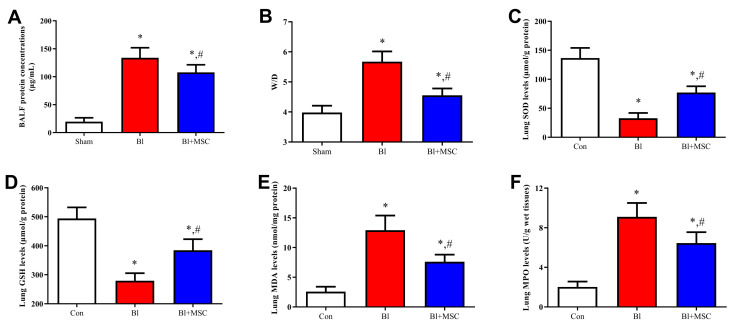
Effects of GMSCs on pulmonary oedema and oxidative stress-related indicators. (**A**) Assessment of total protein in BALF. (**B**) Assessment of the wet/dry ratio in lung tissue. (**C**) Assessment of SOD expression in lung tissue. (**D**) Assessment of GSH content in lung tissue. (**E**) Assessment of MDA content in lung tissue. (**F**) Assessment of MPO expression in lung tissue. * *p* < 0.05 indicates significant differences versus the Con group; ^#^ *p* < 0.05 indicates significant differences versus the Bl group. Values are expressed as the mean ± SEM. Abbreviations: MDA, malondialdehyde; MPO, myeloperoxidase; GSH, glutathione; SOD, superoxide dismutase. White bars indicates Con group, red bars indicates Bl group, blue bars indicate Bl + MSC group.

The concentration of total protein (Figure 11A; *p* < 0.05) and the wet/dry ratio (Figure 11B; *p* < 0.05) were significantly increased after bleomycin instillation, and GMSC intervention caused a significant decrease in the protein concentration (*p* < 0.05) and wet/dry ratio (*p* < 0.05).

### 3.10. Assessment of Oxidative Stress-Related Indicators in Lung Tissue

Bl intervention caused a significant decrease in SOD (*p* < 0.05; Figure 11C) and GSH (*p* < 0.05; Figure 11D) levels and a significant increase in MDA (*p* < 0.05; Figure 11E) and MPO (*p* < 0.05; Figure 11F) levels. Significant downregulation of MDA (*p* < 0.05) and MPO (*p* < 0.05) levels and significant upregulation of SOD (*p* < 0.05) and GSH (*p* < 0.05) levels were detected after GMSC intervention.

### 3.11. Assessment of BALF Cell Count

As shown in Table 2, bleomycin treatment increased the number of total cells (*p* < 0.05) and neutrophils (*p* < 0.05) in BALF, and GMSC intervention attenuated the number of total cells (*p* < 0.05) and neutrophils (*p* < 0.05) in BALF. Bleomycin and GMSCs did not influence the numbers of lymphocytes, macrophages, or eosinophils in BALF.

### 3.12. Assessment of the Levels of Inflammatory Cytokines in BALF

As shown in Table 3, bleomycin instillation increased BALF levels of IL-1β (*p* < 0.05), CXCL-1 (*p* < 0.05), IL-8 (*p* < 0.05), TNF-α (*p* < 0.05), TGF-β (*p* < 0.05), MMP-9 (*p* < 0.05) but decreased BALF levels of IL-10 (*p* < 0.05) compared to Con. GMSC intervention decreased BALF levels of IL-1β (*p* < 0.05), CXCL-1 (*p* < 0.05), IL-8 (*p* < 0.05), TNF-α (*p* < 0.05), TGF-β (*p* < 0.05), and MMP-9 (*p* < 0.05) but increased BALF levels of IL-10 (*p* < 0.05) compared to BL.

### 3.13. Detection of GMSCs Homing in Lung Tissue

To obtain data indicating whether GMSCs migrate to the injured lungs, GMSCs were stained with CM-Dil, and the fluorescence signal was captured with a fluorescence microscope. Engraftment of GMSCs was detected in the lung. Only a very small number of GMSCs were detected in the lung (Supplementary Material 5).

## 4. Discussion

GMSCs were isolated and characterised for the first time in 2009, and GMSCs are rich MSC sources and can be harvested at a low cost [12]. We obtained a novel population of cells from mouse gingival tissue. GMSC characteristics were similar to the properties of bone marrow- and adipose-derived MSCs. GMSCs have a neural crest origin. Hence, GMSCs can differentiate into neural cell lineages [16]. The data of the present study demonstrated that GMSCs could be induced to successfully differentiate into cells of mesodermal and ectodermal origin. These properties provide a scientific basis for the use of GMSCs as promising alternatives for cellular transplantation therapy. The present study reduces the cost of MSC research and contributes to subsequent studies and the application of MSCs.

Mason and Sirius Red staining demonstrated that collagen deposition and the degree of pulmonary fibrosis were increased after bleomycin instillation, and GMSC intervention notably attenuated both of these phenotypes in lung tissue. Our data is consistent with previous conclusions [6,7,8,9,10,11]. Our data demonstrated that GMSCs could be used as a novel tool for bleomycin-induced pulmonary fibrosis. However, the underlying molecular mechanisms through which GMSCs improve bleomycin-induced pulmonary fibrosis have not yet been fully elucidated.

Our data revealed that levels of TGF-β in BALF and gene expression levels of profibrogenic factors (LPA, LPA1, and TGF-β) in lung tissue were significantly increased in response to bleomycin instillation, and GMSCs inhibited these phenomena. Our data suggested that GMSCs exert an antifibrogenic effect by inhibiting LPA, LPA1, and TGF-β expression.

Establishing a hyperinflammatory state is an important mechanism for pulmonary fibrosis [34]. Hence, controlling inflammation is a major part of the treatment of pulmonary fibrosis. Data of the present study demonstrated that GMSC intervention exerts immune effects and suppresses the inflammatory response induced by bleomycin. Our data indicated that bleomycin instillation activated the expression of the proinflammatory factors IL-1β and TNF-α and suppressed the expression of the anti-inflammatory factor IL-10 in lung tissue. GMSCs attenuated gene expression of the proinflammatory factors IL-1β and TNF-α and increased gene expression of the anti-inflammatory factor IL-10. These results demonstrated that GMSCs play vital roles in alleviating pulmonary inflammation.

Neutrophils play a vital role in bleomycin-induced pulmonary fibrosis because neutrophils release profibrotic factors, such as MMP-9 and NE [21,22]. Data of the present study indicated that neutrophil content was significantly increased in BALF and lung, liver and kidney tissues after bleomycin instillation, while GMSC intervention attenuated neutrophil content. Our data demonstrated that GMSC intervention impaired bleomycin-induced MMP-9 and NE release. Therefore, GMSC intervention alleviated bleomycin-induced pulmonary fibrosis by suppressing the expression of MMP-9 and NE. Previous studies have shown that TNF-α, IL-8, and CXCL-1 are chemotactic factors in neutrophils [35,36,37]. Notably, data of the present study indicated that GMSC intervention impairs bleomycin-induced CXCL-1, IL-8, and TNF-α release in lung tissue. Therefore, GMSC intervention inhibits neutrophil infiltration by suppressing CXCL-1, IL-8, and TNF-α expression.

Oxidative stress injury is an important component in the pathogenesis of pulmonary fibrosis [38]. Our data demonstrated that bleomycin administration led to oxidative stress injury in lung tissue and that GMSC intervention reduced the production of inducible MDA and MPO and increased the levels of SOD and GSH in lung tissue. Hence, GMSC intervention alleviates bleomycin-induced pulmonary fibrosis partly by alleviating oxidative stress injury.

Previous work determined that apoptosis was responsible for the development of pulmonary fibrosis [39,40]. However, the protective effects of MSCs on apoptosis in pulmonary fibrosis have been overlooked. Our data demonstrated that GMSC intervention inhibited bleomycin-induced apoptosis in the lung. GMSC intervention exerted a significant protective effect against bleomycin-induced apoptosis.

The data in the present study indicated that deleterious accumulation of neutrophils within remote vital organs leads to collateral tissue damage. Activation of neutrophils is a critical cause of host tissue injury. GMSC intervention reduced neutrophil infiltration in the lung, liver, and kidney tissues. Hence, GMSCs exert immunomodulatory effects and protect against bleomycin-induced multiple organ injury partly because GMSC decreased by decreasing neutrophil content. Inflammatory cells such as neutrophils attack blood vessels first. Inflammatory cell infiltration and increased lung permeability are caused by increased vascular permeability. However, previous work has ignored the effects of bleomycin and MSCs on blood vessels. Our data determined that bleomycin instillation results in marked aortic injury and increased neutrophil infiltration, lung permeability, and pulmonary oedema, which was reversed by GMSC intervention. Hence, GMSCs protected bleomycin-induced pulmonary fibrosis by alleviating vascular injury, including aortic injury. The protective effects of MSCs on aortic injury induced by bleomycin were demonstrated herein for the first time.

Inflammatory cells, such as neutrophils, attack blood vessels, causing increased vascular permeability, which leads to inflammatory cell infiltration and oedema. Marked neutrophil infiltration and oedema in the heart, liver, lung and kidney were observed after bleomycin instillation, which was reversed by GMSC intervention. The present study demonstrated that the inflammatory response caused by bleomycin was systemic and affected multiple organ injuries. GMSCs played a positive role in protecting against bleomycin-induced multiple organ injuries in mice partly because GMSCs alleviated vascular injury and neutrophil-induced inflammation. MSC intervention can improve bleomycin-induced pulmonary fibrosis in mice, and one of the mechanisms involved in this process is linked to an increase in anti-inflammatory, antioxidant and antifibrotic properties.

## Figures and Tables

**Table 1:** Blood biochemistry measurements of liver and kidney damage markers.

Group	Con	Bl	Bl + MSC	*p*
ALT (U/L)	33.82 ± 8.87	124.33 ± 23.78 *	84.27 ± 15.48 ^#^	<0.05
AST (U/L)	119.64 ± 14.96	180.67 ± 26.45 *	130.25 ± 25.31 ^#^	<0.05
Cre (μmol/L)	55.91 ± 8.98	214.21 ± 10.35 *	134.35 ± 15.49 ^#^	<0.05
BUN (mmol/L)	7.99 ± 1.14	13.56 ± 2.31 *	10.59 ± 2.16 ^#^	<0.05

**Note:** Markers of liver damage (ALT and AST) and markers of kidney damage (Cre and BUN) in the serum were measured. * *p* < 0.05 indicates significant differences in the data versus the Con group; ^#^ *p* < 0.05 indicates significant differences versus the Bl group. Values are expressed as the mean ± SEM. Abbreviations: ALT, alanine aminotransferase; AST, aspartate aminotransferase, Cre, creatinine; BUN, blood urea nitrogen.

**Table 2:** Total and differential cell counts in BALF (cells/mL).

Group	Con	Bl	Bl + MSC	*p*
Total cells	1.25 ± 1.86	8.28 ± 2.14 *	6.58 ± 0.94 ^#^	<0.05
Neutrophils	0.03 ± 0.01	7.35 ± 1.23 *	5.84 ± 2.38 ^#^	<0.05
Lymphocytes	0.05 ± 0.03	0.15 ± 0.14	0.15 ± 0.16	ns
Macrophages	1.23 ± 0.14	1.12 ± 0.60	0.86 ± 0.60	ns
Eosinophils	0.01 ± 0.03	0.01 ± 0.03	0.04 ± 0.05	ns

**Note:** The number of total cells, neutrophils, lymphocytes, macrophages, and eosinophils in BALF was measured. * *p* < 0.05 indicates significant differences in the data versus the Con group; ^#^ *p* < 0.05 indicates significant differences versus the Bl group. Values are expressed as the mean ± SEM.

**Table 3:** Levels of inflammatory factors in BALF.

Group	Con	Bl	Bl + MSC	*p*
IL-1β (ng/mL)	12.64 ± 2.14	706.91 ± 113.55 *	563.14 ± 116.98 ^#^	<0.05
CXCL-1 (ng/mL)	24.22 ± 3.15	503.77 ± 56.11 *	348.14 ± 40.33 ^#^	<0.05
IL-8 (ng/mL)	126.9 ± 14.73	513.24 ± 65.21 *	204.91 ± 21.72 ^#^	<0.05
TNF-α (ng/mL)	13.38 ± 3.91	152.31 ± 28.64 *	78.67 ± 18.97 ^#^	<0.05
TGF-β (ng/mL)	21.35 ± 4.86	165.59 ± 43.54 *	98.45 ± 24.68 ^#^	<0.05
MMP-9 (ng/mL)	7.69 ± 0.62	30.11 ± 3.89 *	18.66 ± 0.21 ^#^	<0.05
IL-10 (ng/mL)	20.65 ± 2.36	12.82 ± 1.65 *	35.69 ± 4.21 ^#^	<0.05
NE activity (nmol/mL)	20.33 ± 0.17	64.89 ± 0.71 *	46.55 ± 0.52 ^#^	<0.05

**Note:** Levels of IL-1β, CXCL-1, IL-8, TNF-α, TGF-β, MMP-9, IL-10, and NE activity in BALF were detected. * *p* < 0.05 indicates significant differences in the data versus the Con group; ^#^ *p* < 0.05 indicates significant differences versus the Bl group. Values are expressed as the mean ± SEM. Abbreviations: IL, interleukin; TNF, tumour necrosis factor; CXCL, C-X-C motif chemokine ligand; TGF, transforming growth factor; MMP, matrix metalloproteinase; NE, neutrophil elastase; MPO, myeloperoxidase.

## Data Availability

The datasets used or analysed in the present study are available from the corresponding author on reasonable request.

## Supplementary Materials

The following are available online at https://www.mdpi.com/article/10.3390/ijms23010099/s1.
