# Supplementary material for: Anti-Inflammatory, Antioxidant, and Antifibrotic Effects of Gingival-Derived MSCs on Bleomycin-Induced Pulmonary Fibrosis in Mice
Source: Int J Mol Sci. 2021 Dec 22;23(1):99. doi: 10.3390/ijms23010099 (PMC8745316; doi:10.3390/ijms23010099)
Supplement: Supplementary file 1 [file ijms-23-00099-s001.zip › ijms-1362260-supplementary.pdf]

Supplementary Material 1

Table S1. Medium information for GMSCs.

Complete medium	DMEM/F12, 10% FBS, 5 ng/mL bFGF, 2 mM L-glutamine
Adipocytes inducing medium	DMEM/F12, 10% FBS, 1 mM dexamethasone, 0.5 mM IBMX, 10 mg/mL insulin and 60 mM indomethacin
Osteogenic inducing medium	DMEM/F 12, 10% FBS, 0.5 mM dexamethasone, 10 mM β-glycerophosphate and 50 mg/mL vitamin C
Chondrogenic inducing medium	DMEM/F 12, 10% FBS, 1% ITS, 50 mg/mL L-proline, 0.1 mM dexamethasone, 0.9 mM sodium pyruvate, 50 mg/mL vitamin C and 10 ng/mL TGF-β3
Neurogenic inducing medium	DMEM/F 12, 2% B27, 1% glutamine, 40 ng/ml medium-1 bFGF, 20 ng/ml EGF, 1 μM all-trans-retionic acid, and 100 μm 2-mercapoethanol

Supplementary Material 2

The methods description, including neutrophil content in lung tissue, kidney injury score, liver injury score, lung injury score, aortic intima media-thickness, detection of ALT, AST, BUN, and CRE in serum, BALF cell count, hydroxyproline assay, ashcroft fibrosis score, and TUNEL staining were provided.

Lung histology and IQA

The middle lobe of the right lung was fixed in 10% (wt/vol) formalin and routinely processed in paraffin sections for hematoxylin and eosin staining. Ten fields from each slice were visualized by microscopy (×100). The average values were taken as a modified semi-quantitative histological index of quantitative assessment (IQA) of lung injury using the following criteria: 0, no alveolitis; +1, thickening of the alveolar septum by a mononuclear cell infiltrate, with involvement limited to focal, pleural-based lesions occupying less than 25% of the lung and with good alveolar architecture preservation; +2, more extensive fibrosis involving 26%-50% of the lung and fibrotic regions, mostly extending inward from the pleura and still focal; +3, widespread fibrosis involving 51%-70% of the lung; and +4, widespread fibrosis involving more than 70% of the lung [1,2].

Fibrosis scoring system

The lung fibrosis score, which is based upon the severity and extent of lung fibrosis and not inflammation present in the peribronchial and interstitial tissues, was assessed by one of us

who is a pulmonary pathologist (AY) as previously described [3]. Lungs were assigned a severity score from 0 (no fibrosis) to 4 (severe fibrosis) while the extent of involvement was quantified on a scale of 1 (occasional alveolar duct and bronchiole involvement) to 3 (more than half of the alveolar ducts and respiratory bronchioles involved). The fibrosis score was calculated as the severity (0-4) multiplied by the extent (1-3).

Neutrophil content: By using a 100-point grid of a known area (62,500 μm^2 at 400X magnification) that was attached to the ocular of the microscope, we counted the number of points that were hitting the alveolar tissue and the numbers of neutrophils and positive cells in each field. The cell density was determined as the number of positive cells in each field divided by the tissue area, and this value was expressed as cells/mm^2 [4]. Morphometric measurements were performed in 15 fields for each animal at 400X magnification by an investigator who was blinded to the specific group that was studied.

Ashcroft fibrosis score

After death, each lung tissue was fixed in buffered 4% paraformaldehyde for 24 hr and embedded in paraffin. Ten consecutive longitudinal sections of the lungs were stained with Msason and examined for pulmonary fibrosis. Each successive field was individually assessed for the severity of interstitial fibrosis using the semiquantitative grading system described by Ashcroft et al [5]. The entire lung section was reviewed at a magnification of 100. For each of the 30-35 microscopic fields needed to review the section, a score ranging from 0 (normal lung) to 8 (total fibrosis) was assigned. The mean score of all fields was taken as the fibrosis score of that lung section. The criteria for grading pulmonary fibrosis were as follows. Grade 0 ¼ normal lung; Grade 1: ¼ minimal fibrous thickening of alveolar or bronchial walls; Grade 2-3: ¼ moderate thickening of walls without obvious damage to lung architecture; Grade 4-5: ¼ increased fibrosis with definite damage to lung architecture and formation of fibrous bands or small fibrous mass; Grade 6-7: ¼ Severe distortion of structure and large fibrous areas; honeycomb lung was placed in this category; Grade 8: ¼ total fibrotic obliteration of the field.

TUNEL staining

The DeadEnd Colorimetric TUNEL System assay kit was used according to the

manufacturer's instructions (Promega, Madison, WI). Briefly, paraffin-embedded sections were deparaffinized by immersing slides in xylene and then through graded ethanol washes followed by PBS. Tissue sections were then fixed in 4% paraformaldehyde for 15 minutes and digested with proteinase K (20 mg/ml) for 22 minutes to permeabilize the tissue. The slides were then washed with PBS, refixed in 4% paraformaldehyde for 5 minutes and washed again. In situ nick end labelling of nuclear DNA fragmentation was performed in a humid chamber for 1 hour in the dark at 37℃. A positive control slide was prepared by treating the tissue sections with RNase-free DNase for 10 minutes before the above labelling. A negative control slide was prepared by omitting recombinant terminal deoxynucleotidyl transferase (rTdT) from the above labelling step. The labelling reaction was stopped by immersing the slides in SSC for 15 minutes followed by a PBS wash. Endogenous peroxidases were blocked by immersing the slides in 0.3% hydrogen peroxide in PBS for 5 minutes and then washing with PBS. Colour was developed using DAB. The slides were then mounted using Permount Mounting Medium (Fisher Scientific, Barrington, IL) and observed under a light microscope.

Kidney injury score

Acute tubular damage was assessed via hematoxylin and eosin (HE) staining by using previously described criteria [6]. Acute tubular damage was scored by using a semiquantitative scoring system (0 to 3 points) for each criterion in 20 randomly sampled high-power fields of the cortex per animal (0 = absent, 1 = mild, 2 = moderate, and 3 = strong acute tubular damage).

Liver injury score

Formalin-fixed liver tissues were embedded in paraffin wax, serially sectioned, and then stained with HE. Morphological characteristics (including PMN infiltration, interstitial edema, focal necrosis, and hemorrhage/congestion) were evaluated under a light microscope, and Suzuki scores (i.e., the sum of the scores of congestion [0: none; 4: severe], vacuolization [0: none, 4: severe], and necrosis [0: none; 4: > 60%]) were calculated to determine the liver injury level [7,8].

Aortic intima media-thickness

Ascending thoracic aorta was dissected and rinsed with cold phosphate-buffered saline and placed in 10% neutral buffered formalin until sectioning for microscopic analysis. Aortic specimens were serially sectioned into three or four rings and embedded entirely in tissue blocks for hematoxylin and eosin (HE) staining. Histologic sections were then reviewed by one person (O.T.) and scanned by a computer assisted imaging device (CAS-200 Cell Analysis System; Bacus Laboratory, Chicago, IL, USA) for measurement of the vessel wall thickness. The mean value of the vessel wall thickness from the endothelial surface to the adventitia was recorded from 10 different locations spanning the entire crosssection. The mean values of aortic wall thickness were first normalized for overall body weight, since it has been shown that larger mice generally exhibit larger vessel wall thickness [9].

Detection of ALT, AST, BUN, and CRE in serum

Blood samples were collected and detected the levels of Alanine Aminotransferase (ALT), Aspartate Aminotransferase (AST), blood urea nitrogen (BUN) and creatinine (CRE) using a Fuji Dri-Chem 3500i Biochemistry Analyzer (Fujifilm Ltd, Japan).

BALF cell count

The cell pellets were resuspended in 1 ml of PBS, and the total cell counts were performed using a Neubauer chamber. For differential cells counts, cytospin slides were prepared and stained with Diff-Quick; 300 cells were counted per slide [10,11].

Hydroxyproline Assay

The lung tissues were homogenized in 10 volumes of distilled water. Homogenates were hydrolyzed in 12 M HCl at 120 °C for 3 h as previously described. Hydroxyproline was measured colorimetrically (560 nm) after hydrolysis using an assay kit (Nanjing Jiancheng Bioengineering Institute, Nanjing, China) [12].

Table S2.

Group	Con	Bl	Bl + MSC	P
Lung injury score	0.65 ± 0.07	9.44 ± 1.13*	6.36 ± 0.57#	< 0.01
Aschoft fibrotis score	0.31 ± 0.02	4.56 ± 0.46*	3.11 ± 0.26#	< 0.01
Hydroxyproline (μg/μng)	16.34 ± 1.14	32.56 ± 2.75*	21.97 ± 1.91#	< 0.01
Kidney injury score	0.43 ± 0.03	6.54 ± 0.89*	3.25 ± 0.72#	< 0.01
Liver injury score	0.13 ± 0.01	6.78 ± 0.71*	3.51 ± 0.48#	< 0.01
Aortic intima media-thickness (mm)	49.23 ± 9.12	66.45 ± 13.17*	53.35 ± 11.8#	< 0.01
Aortic-intima media thickness (mm/g of body weight)	1.79 ± 0.14	2.42 ± 0.19*	2.18 ± 0.21#	< 0.01
Destiny of neutrophils in lung tissue(cells/mm^2)	130.22 ± 1.62	2149.87 ± 281.74*	1136.82 ± 135.63#	< 0.01
Airway aollagn (μm^2 collagn/μm^2 tissue)	24.33 ± 2.27	87.35 ± 9.34*	60.75 ± 5.45#	< 0.01
Apoptosis index (%)	8.42 ± 0.92	27.46 ± 2.64*	19.41 ± 1.83#	< 0.01

Notes: Lung injury score, Ashcroft fibrosis score, aortic intima media-thickness, hydroxyproline levels, destiny of neutrophils in lung tissue, airway aollagn, and apoptosis index in the lungs were measured. Liver injury score and kidney injury score were measured. # *P*<0.05 compared with the Con group. * *P*<0.05 compared with the Bl group.

Supplementary Material 3

Table S3. PCR primer sequence information of mouse.

Gene name	Primer sequence
CXCL-1	CTGGGATTCACCTCAAGAACATC
	CAGGGTCAAGGCAAGCCTC
IL-8	CAAGGCTGGTCCATGCTCC
	TGCTATCACTTCCTTTCTGTTGC
TNF-α	CCCTCACACTCAGATCATCTTCT
	GCTACGACGTGGGCTACAG
LPA	AGCCATGAACGAACAACAGTG
	CATGATGAACACGCAAACAGTG
LPA1	TAAGATGGCCTTCTACAACGGC
	CCATACAGGTATTTGACGTGGAG
TGF-β	CTCCCGTGGCTTCTAGTGC
	GCCTTAGTTTGGACAGGATCTG
MMP-9	CTGGACAGCCAGACACTAAAG
	CTCGCGGCAAGTCTTCAGAG
NE	AGCAGTCCATTGTGTGAACGG
	CACAGCCTCCTCGGATGAAG
MPO	AGTTGTGCTGAGCTGTATGGA
	CGGCTGCTTGAAGTAAAACAGG
IL-10	GCTCTTACTGACTGGCATGAG
	CGCAGCTCTAGGAGCATGTG
GAPDH	AGGTCGGTGTGAACGGATTTG
	TGTAGACCATGTAGTTGAGGTCA

Abbreviation: PCR, polymerase chain reaction

Supplementary Material 4

Flow cytometry.

Using an established procedure, GMSCs in the logarithmic phase were dissociated into a single-cell suspension. After counting, 1 × 10^6 cells were transferred to a sterile centrifuge tube. Precooled 70% ethanol was added dropwise to fix the BM-MSCs, and then, the cells were gently prepared as a single-cell suspension, followed by incubation for 12 h. GMSCs were incubated with PBS containing rabbit-derived primary antibodies against aSMA and FSP1. FITC-labeled goat anti-rabbit IgG was incubated with GMSCs in the dark for 60 min. Finally, the GMSCs were subjected to flow cytometry analysis.

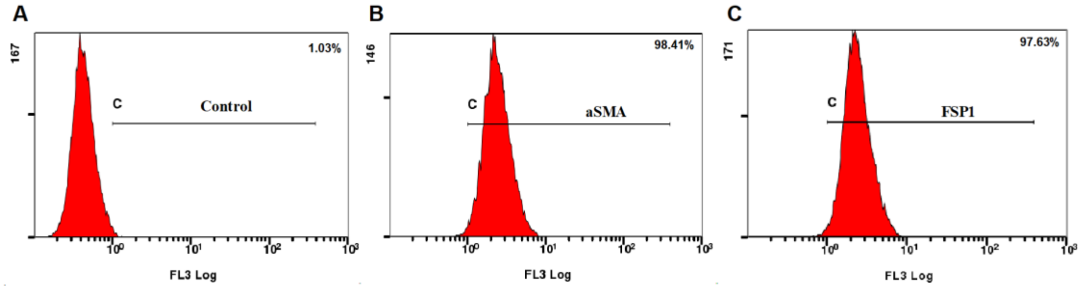

Figure S1. Detection of GMSC surface antigens, including aSMA and FSP1**. Notes:** (A) Control, (B) aSMA, (C) FSP1. The BM-MSCs surface antigens aSMA and FSP1 were positively expressed, as identified by flow cytometry. **Abbreviations:** aSMA, α smooth muscle actin, FSP-1, fibroblast-specific protein 1.

Supplementary material 5

Detection of the homing of GMSCs in lung tissue.

To identify the homing of GMSCs in lung tissue, GMSCs were treated with CM-Dil (C7000, Life Technologies, Eugene, Oregon, USA) for 30 min in an incubator in the dark before GMSC injection. Twenty-four mice were divided into three groups: (1) control (Con) mice received an equivalent volume of normal saline via transtracheal instillation; (2) bleomycin (Bl) mice received 5 mg/kg bleomycin via transtracheal instillation; and (3) bleomycin + MSCs (Bl + MSC) mice received 5 mg/kg bleomycin via transtracheal instillation and 1 × 10^6 GMSCs via transtracheal instillation.

GMSCs were treated with CM-Dil for 30 min. Bl + MSCs mice were treated with 1×10^6 CM-Dil-labelled GMSCs via transtracheal instillation. Frozen lung tissue sections were examined to observe CM-Dil-labelled GMSCs via fluorescence microscopy after mice were sacrificed.

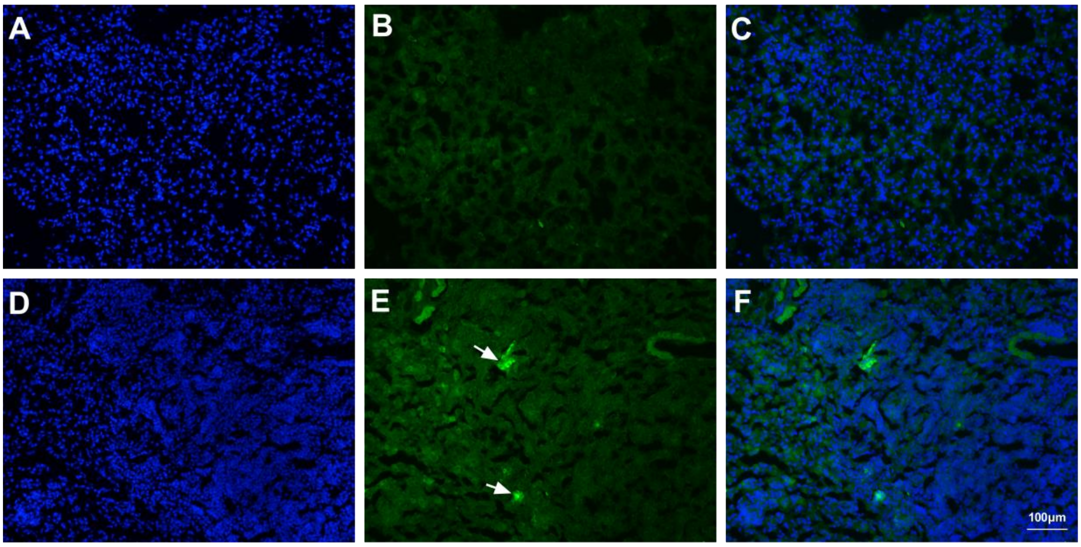

Figure S2. BM-MSCs homing in lung tissue**. Notes:** The CM-Dil-labeled BM-MSCs in the lung were detected. **(A, D)** DAPI, **(B, E)** fluorescence, **(C, F)** Merge. A, B, and C = Bl group. D, E, and F = Bl + MSC group. White arrows indicated CM-Dil-labeled GMSCs. Only a very small percentage of the MSCs were successfully localized in lung tissue. (**A-F, 100x** magnification).

Table S3.

Group	Con	Bl	Bl + MSC	P
Lung injury score	0.65 ± 0.07	9.44 ± 1.13*	6.36 ± 0.57$^{\#}$	< 0.01
Aschoft fibrotis score	0.31 ± 0.02	4.56 ± 0.46*	3.11 ± 0.26$^{\#}$	< 0.01
Hydroxyproline (μg/μng)	16.34 ± 1.14	32.56 ± 2.75*	21.97 ± 1.91$^{\#}$	< 0.01
Kidney injury score	0.43 ± 0.03	6.54 ± 0.89*	3.25 ± 0.72$^{\#}$	< 0.01
Liver injury score	0.13 ± 0.01	6.78 ± 0.71*	3.51 ± 0.48$^{\#}$	< 0.01
Aortic intima media-thickness (mm)	49.23 ± 9.12	66.45 ± 13.17*	53.35 ± 11.8$^{\#}$	< 0.01
Aortic-intima media thickness (mm/g of body weight)	1.79 ± 0.14	2.42 ± 0.19*	2.18 ± 0.21$^{\#}$	< 0.01
Destiny of neutrophils in lung tissue(cells/mm^2)	130.22 ± 1.62	2149.87 ± 281.74*	1136.82 ± 135.63$^{\#}$	< 0.01
Airway aollagn (μm^2 collagn/μm^2 tissue)	24.33 ± 2.27	87.35 ± 9.34*	60.75 ± 5.45$^{\#}$	< 0.01
Apoptosis index (%)	8.42 ± 0.92	27.46 ± 2.64*	19.41 ± 1.83$^{\#}$	< 0.01

Notes: Lung injury score, Ashcroft fibrosis score, aortic intima media-thickness, hydroxyproline levels, destiny of neutrophils in lung tissue, airway aollagn, and apoptosis index in the lungs were measured. Liver injury score and kidney injury score were measured. $^{\#}$ *P*<0.05 compared with the Con group. * *P*<0.05 compared with the Bl group.

Reference

[1] Smith KM, Mrozek JD, Simonton SC, Bing DR, Meyers PA, Connett JE, et al. Prolonged partial liquid ventilation using conventional and high-frequency ventilatory techniques: gas exchange and lung pathology in an animal model of respiratory distress syndrome. Crit Care Med. 1997;25(11):1888-97. doi: 10.1097/00003246- 199711000-00030.

[2] Szapiel SV, Elson NA, Fulmer JD, Hunninghake GW, Crystal RG. Bleomycin-induced

interstitial pulmonary disease in the nude, athymic mouse. Am Rev Respir Dis. 1979;120(4):893-9. doi: 10.1164/arrd.1979.120.4.893.

[3] Cheresh P, Morales-Nebreda L, Kim SJ, Yeldandi A, Williams DB, Cheng Y, et al. Asbestos-induced pulmonary fibrosis is augmented in 8-oxoguanine DNA glycosylase knockout mice. Am J Respir Cell Mol Biol. 2015;52(1):25-36. doi: 10.1165/rcmb. 2014-0038OC.

[4] Lanças T, Kasahara DI, Prado CM, et al. Comparison of early and late responses to antigen of sensitized guinea pig parenchymal lung strips. J Appl Physiol. 2006;100:1610-1616. doi: 10.1152/ japplphysiol.00828.2005.

[5] Yildirim Z, Kotuk M, Erdogan H, et al. Preventive effect of melatonin on bleomycin-induced lung fibrosis in rats. J Pineal Res. 2006;40(1):27-33. doi: 10. 1111/j.1600-079X.2005.00272.x.

[6] Bockmeyer CL, Reuken PA, Simon TP, et al. ADAMTS13 activity is decreased in a septic porcine model. Significance for glomerular thrombus deposition. Thromb Haemost. 2011;105:145-153. doi: 10.1160/TH10-03-0153.

[7] Cay-Huyen Chen, Pei-Shan Tsai, Chun-Jen Huang. Minocycline ameliorates lung and liver dysfunction in a rodent model of hemorrhagic shock/resuscitation plus abdominal compartment syndrome. J Surg Res. 2013;180(2):301-9. doi: 10.1016/j. jss.2012.04.036.

[8] S Suzuki, S Nakamura, T Koizumi, et al. The beneficial effect of a prostaglandin I2 analog on ischemic rat liver. Transplantation. 1991;52(6):979-83. doi: 10.1097/ 00007890-199112000-00008.

[9] Kottarappat N Dileepan, Thomas P Johnston, Yuai Li, et al. Deranged aortic intima-media thickness, plasma triglycerides and granulopoiesis in Sl/Sl(d) mice. 2004;13(5-6):335-41. doi: 10.1080/09629350400008794.

[10] Vieira RP, Claudino RC, Duarte AC, et al. Aerobic exercise decreases chronic allergic lung inflammation and airway remodeling in mice. Am J Respir Crit Care Med. 2007; 176(9):871-7. doi: 10.1164/rccm.200610-1567OC.

[11] Kasahara DI, Poynter ME, Othman Z, et al. Acrolein inhalation suppresses lipopolysaccharide-induced inflammatory cytokine production but does not affect acute airways neutrophilia. J Immunol. 2008; 181(1):736-45. doi: 10.4049/jimmunol. 181.1.736.

[12] Kennedy J, Chandler D, Jackson R, Fulmer J. Reduction in bleomycin-induced lung hydroxyproline content by an iron chelating agent. Chest. 1986;89(3 Suppl):123S-125S. doi: 10.1378/chest.89.3_Supplement.123S.
